# Arabic (Tunisian) translation and validation of the Urogenital Distress Inventory short form (UDI-6) and Incontinence Impact Questionnaire short form (IIQ-7)

**DOI:** 10.1080/2090598X.2019.1678000

**Published:** 2019-10-29

**Authors:** Sameh Ghroubi, Nedra El Fani, Soumaya Elarem, Samar Alila, Houda Ben Ayed, Ons Borgi, Jihen Chmak, Mohamed Habib Elleuch

**Affiliations:** a Department of Physical Medicine and Rehabilitation, Habib Bourguiba University Hospital, University of Sfax, Sfax, Tunisia; b Department of Epidemiology and Community Medicine, Hedi Chaker University Hospital, University of Sfax, Sfax, Tunisia

**Keywords:** Urinary incontinence, Incontinence Impact Questionnaire short form, Urogenital Distress Inventory short form, Arabic (Tunisian) version, translations

## Abstract

**Objective**: To translate and validate an Arabic (Tunisian) version of the Urogenital Distress Inventory short form (UDI-6) and Incontinence Impact Questionnaire short form (IIQ-7), which can be used reliably in daily practice and clinical research for Tunisian and Arabic populations.

**Patients and methods**: This cross-sectional study was conducted from January to June 2018. The UDI-6 assesses the presence of urinary incontinence (UI) and the degree of impairment that it causes, whilst the IIQ-7 evaluates women’s life quality with lower urinary tract symptoms. As UI is a relatively common condition in middle-aged and older women these tools are utilised worldwide. The Arabic (Tunisian) translation and cultural adaptation of the UDI-6 and IIQ-7 was achieved via the forward/backward method and comprehension test within a group of 15 patients. Psychometric validation included testing the questionnaire on a group of 35 patients. Intra-rater reliability was evaluated by calculation of the intraclass correlation coefficient (ICC) for each item of the questionnaires. Cronbach’s α was used to assess internal consistency. The International Consultation on Incontinence Modular Questionnaire short form (ICIQ-SF), in its Arabic version, was used as the ‘gold standard’.

**Results**: For the UDI-6, the ICC was 0.98 demonstrating excellent intra-rater reliability and Cronbach’s α was 0.99 (>0.9), confirming an excellent correlation between the different items. Internal consistency (Cronbach’s α 0.99) and test–retest reliability of the IIQ-7 (ICC 0.98) were very good. For both questionnaires, the κ values for each item ranged from 0.77 to 0.96.

**Conclusions**: We found that the UDI-6 and IIQ-7 questionnaires were valid tools that can be used reliably in daily practice and clinical research for Tunisian and Arabic women with UI.

**Abbreviations**: ICC: intraclass correlation coefficient; ICIQ-SF: Incontinence Modular Questionnaire short form; IIQ-7: Incontinence Impact Questionnaire short form; QoL: quality of life; UDI-6: Urogenital Distress Inventory short form; UI: urinary incontinence

## Introduction

Urinary incontinence (UI) is common in middle-aged and older women, with a reported prevalence varying from 10% to 46% in different populations [1,2]. This is a problem that impairs quality of life (QoL) and can affect working life [3]. To assess the impact of UI in clinical practice and research, we need to properly evaluate the patient’s symptoms using validated questionnaires. These include the self-administered questionnaires the Urogenital Distress Inventory (UDI-6), classified as a grade A recommendation questionnaire [4], and the Incontinence Impact Questionnaire (IIQ-7), in their short forms. These questionnaires make it possible to detect different urogenital symptoms and diagnose the existence or not of UI, the grade of this UI, and its impact on QoL in women who present with it.

The objective of the present study was to validate the UDI-6 and IIQ-7 in the Arabic (Tunisian) language by evaluating the test–retest reliability, internal consistency, and their validity in women with UI in order to provide useful assessment tools for use in Tunisian women with UI.

## Patients and methods

### Patients

The study was conducted in our Department of Physical Medicine and Rehabilitation. The inclusion criteria were: women aged ≥22 years, with LUTS for ≥3 months, and negative urine dipstick results. Exclusion criteria included: neurological diseases (except diabetes neuropathy), malignant tumours, and previous pelvic organ surgery if it occurred within 3 months before the baseline visit, pelvic inflammatory disease, and psychiatric diseases. All patients who were consecutively referred to the Department from January to June 2018 who fulfilled the inclusion criteria, as above, were enrolled.

The Regional Ethics Committee approved the study and all participants provided written informed consent.

### Questionnaires and scoring

The sociodemographic characteristics of the women recruited for the study were collected. The rest of the study variables correspond to the specific questions of the questionnaires as follows: the UDI-6 questionnaire consists of six questions using an ordinal scale with possible answers being: ‘no’, ‘a little’, ‘moderately’, ‘very much’; whilst the IIQ-7 questionnaire is comprised of seven questions defined on the same ordinal scale. For the total score, both of the IIQ-7 and the UDI-6, a value of zero is assigned for the answer ‘no’’, 1 for ‘a little’, 2 for ‘moderately’, and 3 for ‘very much’. Thus, once the values obtained from each of the questions are summed, the total score ranges from zero to 21 for the IIQ-7 and from zero to 18 for the UDI-6. Higher scores indicate more severe symptoms and lower QoL. Raw scores for each item are then transformed, which gives a score ranging from zero to 100 for each item [3].

For comparing methods, we used variables taken from the questions of the International Consultation on Incontinence Modular Questionnaire short form (ICIQ-SF) as the ‘gold standard’. The ICIQ-SF assesses both urogenital symptoms (in the first two questions) and QoL (in the last question), and it has demonstrated good psychometric properties [5]. However, the UDI-6 and IIQ-7 questionnaires give more detailed information, providing, in addition to the classification of the type of UI, the grade at which this occurs. For this, we compared the total score of the ICIQ-SF to the total score of both questionnaires, and we also compared the sum of the first two questions of ICIQ-SF to the UDI-6, and the score of the last question of ICIQ-SF to the IIQ-7.

To assess intra-observer reliability, an interval of 15 days between administration of the UDI-6 and IIQ-7 questionnaires was employed. At the first administration of these questionnaires the ICIQ-SF was also administered in order to test the reliability between methods.

### Translation

Translation into Arabic was carried out in accordance with the forward/backward method and independently performed by three bilingual translators. The translations were then submitted for criticism to three experts (two doctors in physical medicine and one professional translator) to test their intelligibility and to provide the adaptations necessary from a linguistic, as well as a cultural standpoint. A synthesis of the different translations yielded a definitive Arabic version, which was quite similar to the original version, and written in simple language that even those with limited literacy would easily understand. The Tunisian dialect was mentioned in parentheses after some terms that were considered somewhat difficult to understand for a proportion of our population. A backward translation from Arabic into English was then performed by two other professional translators who had not been informed about the desired characteristics of their instrument; the backward translations were subsequently compared and contrasted with the original versions. A preliminary test was also conducted in a group of 15 women with LUTS in order to study the acceptability and understanding of the translated scales. The final versions were then reviewed by the committee of experts in order to implement any necessary adaptations (Appendices 1 and 2).

### Statistical analysis

First, descriptive statistics were calculated for each of the items and the total scores of both questionnaires. Then, internal consistency of the UDI-6 and IIQ-7 were assessed by means of the Cronbach’s α coefficient; values >0.7 were considered acceptable. The agreement between the two observations (test–retest reliability) of the same individual was assessed with the intraclass correlation coefficient (ICC) for total scores or κ index for those categorical items; values >0.7 indicate satisfactory reliability.

Finally, the convergent validity of the UDI-6 and IIQ-7 was estimated by Spearman’s correlation, respectively, with the sum of the answers to the first two questions and the last question of the ICIQ-SF in its Arabic version used as the gold standard. The total score of the ICIQ-SF was also compared with both questionnaires.

## Results

The final sample for analysis of the reliability of the UDI-6 and IIQ-7 questionnaires comprised 35 women, with a mean (SD) age of 54.31 (11.11) years.

Response rates given to each of the questions in the UDI-6 questionnaire, both at the first and second administration were very similar, as shown in Table 1. We also found a good match and internal consistency (Cronbach’s α 0.973) for all items.

**Table 1.** UDI-6.

	UDI-6 responses, *n* (%)					
	No	Little	Moderate	Very much	UDI-6 first and second administrations	κ
**First administration**						
Need for urinary frequency	5 (14)	4 (11.4)	17 (48.5)	9 (25)	Need for urinary frequency	0.95
Sense of urgency	3 (8.5)	4 (11.4)	13 (37.14)	15 (42.8)	Sense of urgency	0.91
Leakage with activity	3 (8.5)	4 (11.4)	13 (37.14)	15 (42.8)	Leakage with activity	0.82
Leakage in small amounts	9 (25)	14 (40)	8 (22.85)	4 (11.4)	Leakage in small amounts	0.95
Difficulty emptying	21 (60)	6 (17.14)	4 (11.4)	4 (11.4)	Difficulty emptying	0.95
Pain or discomfort	7 (20)	18 (51.42)	7 (20)	3 (8.5)	Pain or discomfort	0.72
**Second administration**						
Need for urinary frequency	5 (14)	4 (11.4)	18 (51)	8 (22.8)		
Sense of urgency	3 (8.5)	4 (11.4)	13 (37)	15 (42.8)		
Leakage with activity	3 (8.5)	3 (8.5)	16 (45.7)	13 (37.1)		
Leakage in small amounts	9 (25.7)	15 (42.85)	7 (20)	4 (11.4)		
Difficulty emptying	20 (57)	7 (20)	4 (11.4)	4 (11.4)		
Pain or discomfort	8 (22.8)	20 (57)	5 (14)	2 (5.7)		

As for the percentage of each of the responses obtained through the questions of the ICIQ-SF, 12 women (34.28%) had leaks 2 or 3 times a week, and 17 (48.57%) a very small amount of leaks.

In terms of QoL, where a score of zero indicates no involvement and 10 much involvement, we found that most of the patients (54.28%) had poor QoL with a score between 1 and 6.

The results obtained for the ICC indicated that the correlation between the first and second administration of the UDI-6 questionnaire was very good (ICC 0.98).

The correlation between the first administration of the UDI-6 and the total score of the ICIQ-SF and between this first administration and the sum of the first two questions of the ICIQ-SF was significant and moderate, with a Spearman’s coefficient of 0.54 and 0.6, respectively.

For the IIQ-7, both at the first and second administration, similar data were obtained (Table 2). Between the first and second administration of the IIQ-7 there was very good agreement (ICC 0.99).

**Table 2.** IIQ-7.

	IIQ-7 responses, *n* (%)					
	No	Little	Moderate	Very much	IIQ-7 first and second administration	κ
**First administration**						
Ability to do household chores	9 (25.7)	11(31.42)	9 (25.7)	6 (17.14)	Ability to do household chores	0.88
Ability for physical activity	9 (25.7)	8 (22.85)	13 (37.14)	5 (14)	Ability for physical activity	0.92
Recreational activities	10(28.5)	6 (17.14)	12 (34.28)	7 (20)	Recreational activities	0.80
Ability to travel	8 (22.85)	8 (22.85)	14 (40)	5 (14)	Ability to travel	0.82
Participation in social activities	14 (40)	8 (22.85)	10 (28.57)	3 (8.57)	Participation in social activities	0.92
Emotional health	4 (11.4)	11 (31.42)	9 (25.7)	11 (31.42)	Emotional health	0.96
Frustration	6 (17.14)	8 (22.85)	11 (31.42)	10 (28.57)	Frustration	0.88
**Second administration**						
Ability to do household chores	8 (22.8)	10 (28.5)	11 (31.42)	6 (17.14)		
Ability for physical activity	7 (20)	10 (28.5)	13 (37.14)	5 (14)		
Recreational activities	10 (28.5)	8 (22.85)	13 (37.14)	4 (11.4)		
Ability to travel	6 (17.1)	10 (28.5)	14 (40)	5 (14)		
Participation in social activities	14 (40)	11 (31.4)	7 (20)	3 (8.57)		
Emotional health	3 (8.57)	12 (34.2)	9 (25.71)	11 (31.42)		
Frustration	6 (17.14)	7 (20)	14 (40)	8 (28.85)		

It should be mentioned that the question on emotional health obtained the highest score when evaluating the correlation between the first and second administration, reaching values within the threshold at which the κ index indicated very good agreement. For all other questions in the IIQ-7, although κ values were below those of the emotional health question, they were all >0.7, which indicated a good or very good agreement for all of them (Table 1). For this questionnaire, very good internal consistency was obtained with a Cronbach’s α of 0.99.

On the other hand, the correlation between the total score of the IIQ-7 and total ICIQ-SF was significant but weak, showing a Spearman’s coefficient of 0.37. However, when considering only the score of the last question of the ICIQ-SF, which assesses the impact on QoL, the correlation was stronger with a Spearman’s coefficient of 0.9.

## Discussion

In the present study, we performed psychometric validations of Arabic translated and Tunisian-adapted versions of the IIQ-7 and UDI-6. Our present results showed that these versions were psychometrically strong and could be used to reliably and accurately measure urogenital symptoms and QoL of women with UI in Arab Countries and particularly in Tunisia.

The two specific questionnaires, the UDI-6 and IIQ-7, have shown promising utility for the evaluation of symptoms distress and health-related QoL. They have also proved significant in characterising the diverse kinds of established UI [6].

The translation process was organised in two steps: the actual translation of the items and the adaptation of the questionnaire to the cultural context and the features of our target population.

Given that there is no consensus in translation methodology [7], we chose to adopt the forward/backward translation method. We used easy literary Arabic terms because literary Arabic is the official language that unifies many countries.

The Tunisian dialect was used in parentheses to explain and simplify the technical and difficult words and to make the questionnaire culturally well fitted to the Tunisian population, where the rate of illiteracy is in two figures in people aged >30 years and is >40% in people aged >60 years [8].

The preliminary test allowed us to detect the terms that might be poorly or not at all assimilated by patients, in particular those with limited literacy, allowing for any adaptations with an initial assessment of the scales.

Several preliminary studies are sometimes needed, in order to produce usable questionnaires [9]. The inclusion of a volunteer patient helped to identify comprehension difficulties and the potential sources of confusion within the items. The cultural adaptation of the questionnaires changed neither the number of items nor their pattern.

As to the metrological qualities of these questionnaires, our present results revealed that the Arabic versions of the UDI-6 and IIQ-7 were reliable for use in women with UI. There was high internal consistency (Cronbach’s α > 0.9) for both the UDI-6 and IIQ-7, which is comparable to the original version and other translated versions [6,10,11], and generally considered good when the Cronbach’s α coefficient value is >0.7.

We studied the test–retest reliability using the ICC. An ICC >0.5 is considered sufficient and suggested satisfactory repeatability of the Arabic-language version of the questionnaire [6].

High values of ICC are consolidated with high κ index values, which showed a very good reproducibility of each of the questions that comprised the UDI-6 and IIQ-7 questionnaires, which, in turn, had a good internal consistency. These results were comparable, for both questionnaires, to those of the Spanish versions [3].

To evaluate the convergent validity, we used the ICIQ-SF as the gold standard, comparing between the total score of the UDI-6 and the sum of the answers to the first two questions of the ICIQ-SF, and the total score of the IIQ-7 with the last question of the ICIQ-SF, calculating Spearman’s correlation. The total score of the ICIQ-SF was also compared to both questionnaires.

The moderate correlation found by comparing the total score of the UDI-6 with this gold standard could be explained by the fact UDI-6 questionnaire provides more detailed urogenital symptoms information and classification of the type UI and the degree at which this occurs, this result was comparable to that of the Spanish version [3].

On the other hand, the weak correlation between the total score of the IIQ-7 and the total score of the ICIQ-SF (Spearman’s coefficient 0.37) could be explained by the fact that the impact of UI on QoL was evaluated just in the last question of the ICIQ-SF. This was confirmed by the high correlation between the total score of the IIQ-7 and the last question of ICIQ-SF (Spearman’s coefficient 0.93).

Both questionnaires were easy and quick to administer to the women, and therefore potentially useful instruments for assessing UI and its impact on QoL in Arabic women. As there are two questionnaires, they can be more specific and nuanced than, for example, the gold standard used, which includes both concepts in one questionnaire.

The present study was conducted only in women and the sample size was small, as female UI is still considered a taboo in our country and not talked about or discussed in public. The present study was conducted in a referral department and therefore the symptoms of the group of patients may be more severe and thus may limit generalisation. However, the present study has several strengths, such as the adaptation of this Arabic version to the Tunisian population by including the Tunisian dialect in parentheses to explain difficult Arabic words and to make the questionnaire well fitted to our target population.

## Conclusion

Our present study showed that the Arabic version and the Tunisian adaptation of the UDI-6 and the IIQ-7 were reliable and valid instruments to assess UI and its impact on QoL in Tunisian women.
